# Structural modifications in Bermuda grass [*Cynodon dactylon* (L.) Pers.] ecotypes for adaptation to environmental heterogeneity

**DOI:** 10.3389/fpls.2022.1084706

**Published:** 2023-01-19

**Authors:** Aasma Tufail, Farooq Ahmad, Mansoor Hameed, Muhammad Ahsan, Mohammad K. Okla, Umme Habibah Siddiqua, Noreen Khalid, Madiha Rashid, Anis Ali Shah, Momtaz M. Hegab, Hamada AbdElgawad

**Affiliations:** ^1^ Department of Botany, Division of Science and Technology, University of Education, Lahore, Pakistan; ^2^ Department of Botany, University of Agriculture, Faisalabad, Pakistan; ^3^ Department of Horticultural Sciences, Faculty of Agriculture & Environment, The Islamia University of Bahawalpur, Bahawalpur, Pakistan; ^4^ Botany and Microbiology Department, College of Science, King Saud University, Riyadh, Saudi Arabia; ^5^ Department of Chemistry, University of Jhang, Jhang, Pakistan; ^6^ Department of Botany, Government College Women University, Sialkot, Pakistan; ^7^ Research Institute of Medicinal and Aromatic Plants, Beni-Suef University, Beni-Suef, Egypt; ^8^ Integrated Molecular Plant Physiology Research, Department of Biology, University of Antwerp, Antwerpen, Belgium

**Keywords:** ecotypes, environmental heterogeneity, sclerification, trichomes, water conservation

## Abstract

**Introduction:**

It is well known that different ecotypes adopt different mechanisms to survive under environmental stress conditions. In this regard, each ecotype showed different type of modifications for their existence in a specific habitat that reflects to their ecological success.

**Methods:**

Here, differently adapted ecotypes of Bermuda grass [Cynodon dactylon (L.) Pers.] were collected to evaluate their differential structural and functional modifications that are specific to cope with environmental stress conditions. The soil that adheres ecotypes roots were highly saline in case of DF-SD (Derawar Fort-Saline Desert), UL-HS (Ucchali Lake-Hyper Saline) and G-SSA (Gatwala-Saline Semiarid) ecotypes. Soils of S- HS (Sahianwala-Hyper Saline), S-SW (Sahianwala-Saline Wetland) and PA-RF (Pakka Anna-Reclaimed Field) were basic (pH 9 to 10). Soils of UL-HS and PA- HS (Pakka Anna-Hyper Saline), KKL-S (Kalar Kahar Lake-Saline), BG-NS (Botanic Garden-Non Saline) and G-SSA were rich in organic matter, and soil of BG-NS and DF-SD were rich in minerals. Anatomical modifications were performed by using the free hand sectioning technique and light microscopy.

**Results and Discussion:**

DF-SD is one of the best ecotypes which showed anatomical modifications to cope with environmental changes. These modifications included stem cross-sectional area and leaf sheath thickness that contribute towards water storage, vascular tissues for proficient translocation of solutes and trichomes that provide resistance to water loss. On the other hand, sclerification in root is the only notable modification in the Gatwala Saline Semiarid (G-SSA) ecotype from saline arid habitat where rainfall is not as low as in the Cholistan Desert. Two ecotypes from hyper-saline wetlands, UL-HS and KL-HS showed increased number and size of vascular tissue, central cavity and sclerification in stem which are important for solutes conduction, water loss and salts bulk movement, respectively. The ecotype from reclaimed site was not much different from its counterpart from hyper-saline dryland. Overall, anatomical modifications to maintain water conservation are key mechanisms that have been identified as mediating stress tolerance in C. dactylon ecotypes.

## Introduction

Plants survival in various environmental conditions depends on morphological, physiological and anatomical adaptive modifications (Jia et al., 2021), which is often genetically fixed during evolutionary history under a particular set of environments (Hoffmann and Willi, 2008). Such adaptive characteristics have been reported in many species or even populations/ecotypes of same species, for example *Cenchrus ciliaris*, *Elymus nutans*, *Phragmites karka* and *Sporobolus virginicus* (Abideen et al., 2015; Mansoor et al., 2019; Qi et al., 2020). It is also evident that different ecotypes responded differently or adopt different mechanisms for their survival under environmental stresses (Mansoor et al., 2019; Sarwar et al., 2022).

Anatomical modifications are strongly responsive to environmental factors and greatly help in the identification of plant species, cultivars and populations of same species (Valladares et al., 2014; Wyka et al., 2019). These anatomical modifications can minimize detrimental effects of environmental stresses (Poljakoff-Mayber, 1988). For instance, plant capacity to maintain high water potential was largely explained by the anatomical modifications including cuticular composition, greater leaf thickness, and stomatal behavior that were associated with increased resistance to water flux (Bolger et al., 2014), Thus structural modifications are important to assess degree of tolerance in plant species under variety of environmental stresses in addition to other physiological and biochemical processes (Moray et al., 2015). In this context, structural development and modification are crucial in the survival and growth of plants under harsh climatic conditions (Li et al.2022; Li et al., 2021). Modifications such as pubescence or salt hairs and glands (Ahmad et al., 2016), aerenchyma formation (Watson et al., 2015), succulence, stomatal size, density and orientation, bulliform cells (Manokari et al., 2021; Iqbal et al., 2022) and high plasticity vessel numbers and lumina contribute to the prevailing water availability (Beniwal et al., 2010). Moreover, efficient water-conducting vessels (Al Hassan et al., 2015) and intensive sclerification are critical for water conservation and salt tolerance in saline, hot and arid environments (Tanase et al., 2016).

*Cynodon dactylon* (L.) Pers. is extensively grown worldwide and is widely distributed in almost all environmental conditions (Shumail et al., 2022), but more recently, the area of interest is low freezing temperature that can affect its distribution (Chen et al., 2015). *C. dactylon* tolerance to environmental stresses has been well documented in many studies such as salinity (Yin et al., 2022), aridity (Akram et al., 2015), freezing (Hu et al., 2016), high temperature (Yu et al., 2015), high altitudes (Aćić et al., 2015) and waterlogging (Yuan et al., 2022). Ecotypic variability in this C_4_ perennial grass is tremendously high (Casler, 2022; Schiavon et al., 2016) and that might be the reason of occupying a variety of habitats including river and canal beds (Chirebvu and Chimbari, 2015), forests (Joubert et al., 2015), wastelands (Nowak et al., 2016), prairies, savannas and grasslands (Radutoiu, 2015).

Plants incorporate genetically-fixed adaptive features to cope with environmental changes (Hufbauer et al., 2015; Bachofen et al., 2021). At the anatomical level, several plant traits have been identified. However, understanding how trees acclimate to alkaline and saline soils and to natural fluctuations in environmental stress requires further research. Therefore, field trials were performed to investigate the evaluation differential structural and functional modifications that are specifically of ecological significance. We hypothesized that differently adaptive populations might have some specific adaptations to cope with environmental heterogeneity to which they are exposed.

## Material and methods

### Collection sites

**Figure 1:**
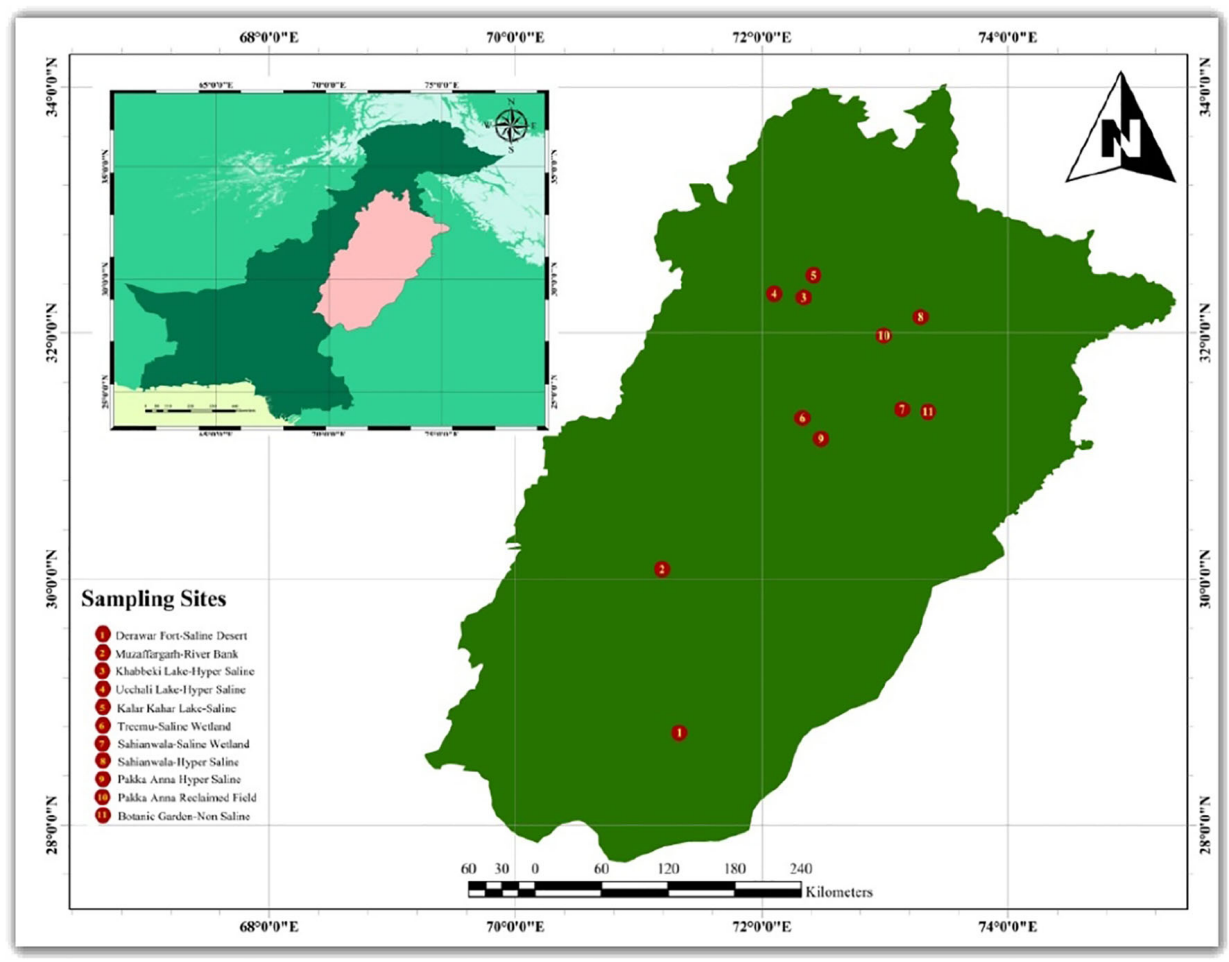
Map of the Punjab, Pakistan showing collection sites of Cynodon dactylon ecotypes.

Various ecotypes of [*Cynodon dactylon* (L.) Pers.] were selected from the Punjab, Pakistan (**Figure 1**) from different ecological conditions (**Table 1**). The ecotypes, DF-SD (Derawar Fort-saline desert), S-HS (Sahianwala-Hyper Saline) and G-SSA (Gatwala-Saline Semiarid) were selected from saline arid regions. Ecotypes KL-HS (Khabbeki Lake-Hyper Saline), UL-HS (Ucchali Lake-Hyper Saline) and KKL-S (Kalar Kahar Lake-Saline) were from saline lakes in the Salt Range. Ecotypes S-SW (Sahianwala-saline wetland) and T-SW (Treemu-saline wetland) were from saline waterlogged areas. Two ecotypes were from salt-affected wasteland, PA-SW (Pakka Anna from Hyper saline wasteland) and PA-RF (Pakka Anna- Reclaimed field). Two ecotypes were from non-saline well moist habitats, MG-RB from Muzaffargarh-river bank and BG-NS from Botanic Garden-non saline habitat.

**Table 1:** Soil physio-chemical characteristics of the selected habitats from Punjab, Pakistan.

Habitats	E.C (dS m^-1^)	pH	Organic matter (%)	Available P (mg Kg^-1^)	Saturation (%)	Na^+^ (mg Kg^-1^)	Ca^2+^ (mg Kg^-1^)	K^+^ (mg Kg-1)	Mg^2+^ (mg Kg^-1^)	Cl^-^ (mg Kg^-1^)
DF-SD	25.1k	7.7cd	0.1c	16.0i	60.0d	4253.4k	64.9g	288.2j	0.5b	2351.9k
M-RB	2.9c	7.5ab	1.2ef	9.7de	58.0d	320.4c	16.5a	61.7c	0.4a	320.5c
KL-HS	11.5h	7.8d	1.3gh	10.0ef	60.0d	2336.7h	37.1cd	121.0g	0.4a	1086.7h
UL-HS	19.4j	7.4a	1.4h	15.8i	60.0d	4035.1j	44.9e	160.5i	0.4a	2021.6j
KKL-S	5.1e	7.8d	1.0d	10.8ef	34.0bc	1052.9f	56.2f	111.9f	0.4a	471.7e
T-SW	12.5b	7.6bc	1.1de	12.0g	38.0c	2895.2b	44.9e	132.5h	0.5b	1144.8a
S-SW	13.3g	9.9h	0.6b	14.2h	34.0bc	2719.7i	33.8c	56.1bc	0.5b	1229.7g
S-HS	4.4d	10.2i	0.3a	7.1b	28.0a	947.2e	29.1bc	83.7d	0.4a	446.8d
PA-HS	6.7f	8.6f	1.4h	8.1bc	38.0c	1320.6g	37.1cd	91.1e	0.4a	656.2f
PA-RF	2.2b	9.6g	0.3a	8.8cd	31.0ab	461.7d	44.8e	88.4de	0.5b	232.7b
BG-NS	1.0a	8.4e	1.3gh	16.1i	30.0ab	81.6a	40.5d	13.2a	0.4a	312.2c
G-SSA	19.3j	7.7cd	1.3gh	1.3a	31.0ab	4107.7j	27.9b	51.8b	0.5b	1823.7i

DF-SD, Derawar Fort-saline desert; M-RB, Muzaffar garh-River bank; KL-HS, Khabbeki Lake-hyper saline; UL-HS, Ucchali Lake-hyper saline; KKL-S, Kalar Kahar Lake-saline; T-SW, Treemu-wetland; S-SW, Sahianwala-saline wetland; S-HS, Sahianwala-hyper saline; PA-HS, Pakka Anna-hyper saline; PA-RF, Pakka Anna-reclaimed field; BG-NS, Botanic Garden-non saline; G-SSA, Gatwala-saline semiarid.

### Soil analysis

The soil that adheres the roots was taken (from 16 cm depth) from each habitat to analyze the physico-chemical characteristics (**Table 1**). Soil pH and EC were measured by pH/EC meter (WTW series InoLab pH/Cond 720). Sodium (Na^+^), potassium (K^+^) and calcium (Ca^2+^) contents were determined on flame photometer (Jenway, PFP-7), whereas Cl^─^ contents on chloride meter (Model 926; Sherwood Scientific Ltd., Cambridge, UK), Available phosphorus in soil was determined by the method of Bray and Kurtz (1945) and magnesium (Mg^2+^) was determined by the method of Richards (1968) with an atomic absorption spectrophotometer (Model Analyst 3000; PerkinElmer, Norwalk, CT).

### Light microscopy analysis

Permanent slides of Transvers sections of root, stem, leaf sheath and leaf blade were prepared by free-hand sectioning technique. Series of ethanol for dehydration of transverse sections and a standard double-satining (safranin and fast green) procedure were used by Sass (1951).

Photographs were taken by a camera-equipped light microscope (Meiji Techno: MT4300H USA). 

### Statistical analysis

The data were subjected to analysis of variance in completely randomized design with factorial arrangement and three replications. The data were also subjected to redundancy analysis using Conoco 4.5 computer software.

## Results

### Soil physico-chemical characteristics

Soil at DF-SD was highly saline where ECe 25.1 dS m^-1^ was recorded with Na^+^ concentration 4253.4 mg Kg^─1^ and Cl^─^ 2351.9 mg Kg^─1^. ECe at two other highly saline sites, UL-HS and G-SSA, was over 19 dS m^-1^, where Na^+^ was over 4000mg Kg^-1^ and Cl^─^ over 2000mg Kg^-1^ (**Table 1**). Soil of S-HS was strongly basic with pH 10.1 whereas other two sites S-SW and PA-RF also have basic soil with pH about 9. The maximum organic matter was recorded in the soils of UL-HS and PA-HS with (1.4%) whereas at other three sites KL-HS, BG-NS and G-SSA, it was 1.3%. The maximum available P (16.1 mg Kg^-1^) was recorded at BG-NS, which was followed by that in DF-SD. The maximum Ca^2+^ (288.2 mg Kg^-1^) and K^+^ concentration (64.9 mg Kg^-1^) was recorded at DF-SD. There was a little variation in soil Mg^2+^at different study sites (**Table 1**).

### Root microscopic analysis

**Figure 2:**
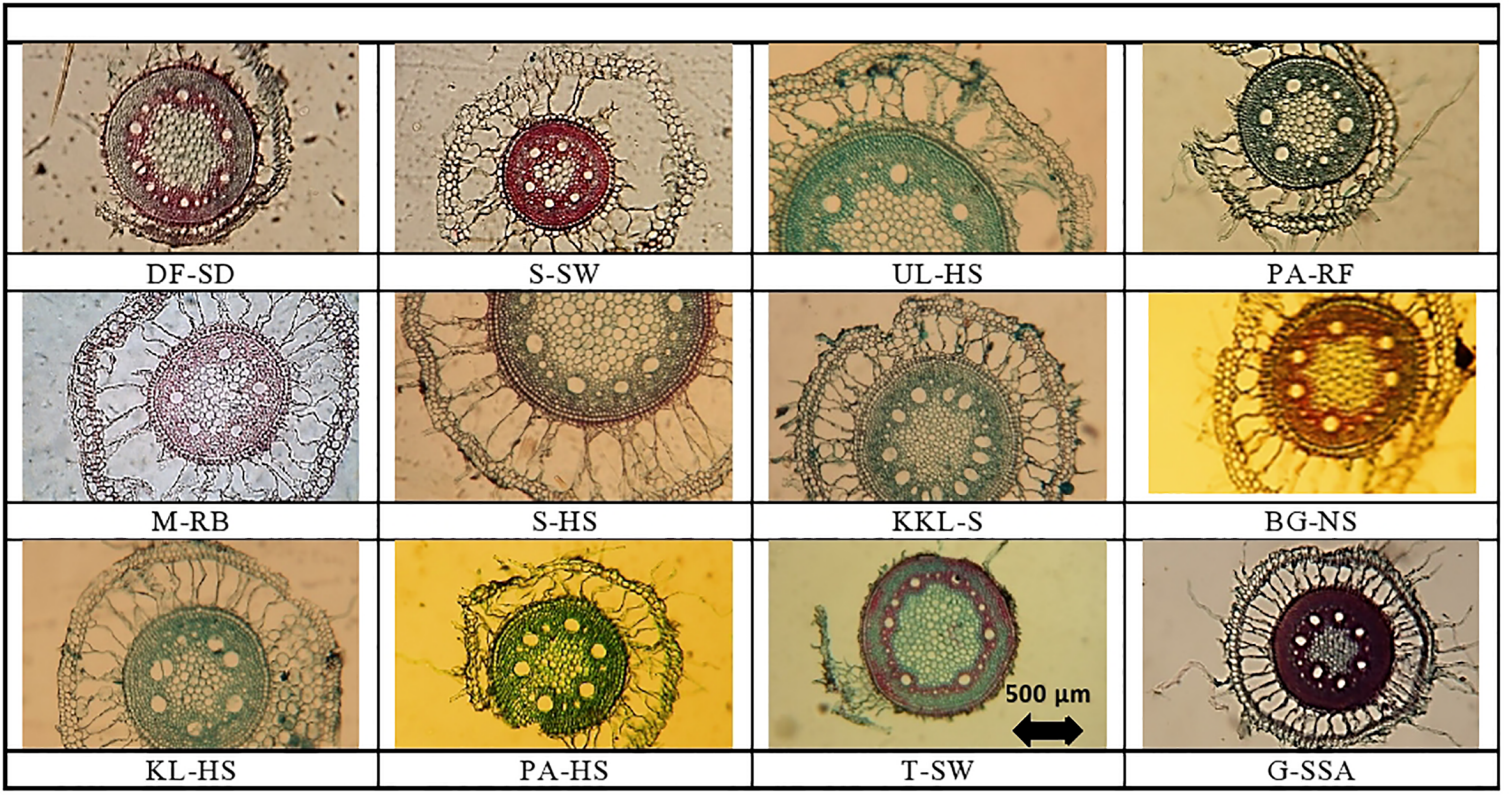
Root tranverse sections of Cynodon dactylon ecotypes collected from the Punjab, Pakistan DF-SD, Derawar Fort-Hyper Desert; M-RB, Muzaffar Garh-River Bank; KL-HS, Khabbeki Lake-Hyper Salin; UL-HS,Ucchali Lake-Hyper Saline; KKL-S; Kalar Kahar Lake-Saline; T-SW, Treemu-Saline Wetland; S-SW, Sahianwala-Saline Wetland; Sahianwala-Hyper Saline; PA-HS, Pakka Anna-Hyper Saline; PA-RF, Pakka Anna-Reclaimed Field; BG-NS, Botanic Gardine-None Saline; G-SS, Gatwala-Saline Semiarid.

Variation regarding anatomical characteristics was exceedingly high. The thickest roots were from UL-HS, which were significantly higher than the second best from S-HS (**Table 2**; **Figure 2**), and the thinnest root were recorded from BG-NS. The maximum epidermal cell area was recorded in KKL-S, followed by that in S-HS. The minimum value was recorded in the T-SW that was significantly lower than the second minimum from M-RB.

**Table 2:** Root Anatomical characteristics of the selected habitats from Punjab, Pakistan.

Habitats	Root cross-sectional area (mm^2^)	Epidermal cell area (µm^2^)	Cortex region thickness (µm)	Cortical cell area (µm^2^)	Aerenchymatous area (µm^2^)	Endodermal cell area (µm^2^)	Metaxylem area (µm^2^)	Phloem area (µm^2^)	Pith area (µm^2^)	Pith cell area (µm^2^)
DF-SD	2.1b	1011.5c	174.1a	2197.3a	30797.4d	104.6a	8719.6f	42.4c	188170.9e	4429.5f
M-RB	3.2d	941.7b	571.2f	5214.3f	64367.7i	837.1f	7934.8e	52.3d	130674.2d	4900.4g
KL-HS	4.0e	1447.4e	508.6e	10132.1j	60932.2h	1203.3h	16009.1j	52.3d	130674.2d	4237.7f
UL-HS	9.0f	1586.9f	552.2f	6278.1h	64088.6i	906.8g	16567.1j	52.3d	522696.9g	7586.0h
KKL-S	4.7e	2249.6i	432.5d	8893.9i	44330.1f	209.3b	9713.6h	42.4c	130674.2d	2546.1b
T-SW	2.8c	627.8a	348.2c	4708.6e	13619.9a	348.8c	4481.9b	42.4c	256121.5f	3644.8e
S-SW	3.5d	1726.5g	568.5f	23019.6k	48131.9g	470.9d	3295.9a	52.3d	47042.7a	2232.2b
S-HS	7.0e	2127.6h	693.6g	5859.5g	124689.5j	976.6g	13375.9i	42.4c	522696.9g	12678.2
PA-HS	2.7c	1046.4c	353.6c	3365.7c	39970.4e	871.9f	13672.2i	42.4c	83631.5b	2301.9b
PA-RF	2.4b	1499.8e	307.4b	4429.5d	32872.9d	627.8e	9207.8g	42.4c	101194.1c	3173.9d
BG-NS	1.5a	1185.9d	356.3c	2790.3b	22496.4c	313.9c	5528.2d	33.4b	101194.1c	2877.5c
G-SSA	2.1b	1220.7d	348.2c	2214.8a	17840.2b	279.0b	4970.1c	33.7b	83631.5b	1430.0a

DF-SD, Derawar Fort-saline desert; M-RB, Muzaffar garh-River bank; KL-HS, Khabbeki Lake-hyper saline; UL-HS, Ucchali Lake-hyper saline; KKL-S, Kalar Kahar Lake-saline; T-SW, Treemu-wetland; S-SW, Sahianwala-saline wetland; S-HS, Sahianwala-hyper saline; PA-HS, Pakka Anna-hyper saline; PA-RF, Pakka Anna-reclaimed field; BG-NS, Botanic Garden-non saline; G-SSA, Gatwala-saline semiarid.

Cortical region thickness varies from 693.6 µm^2^ at S-HS to 174.1 at DF-SD site. Thick cortical region was also recorded in the ecotypes from three sites (M-RB, UL-HS and S-SW), which were over 550 µm^2^.Variation in cortical cell area was tremendously high and the maximum cortical cell area was recorded in the ecotypes from S-SW, which was about two-fold greater than the second maximum KL-HS. The minimum of this parameter was recorded in the ecotype from DF-SD. Endodermal cell area was the maximum in ecotype from KL-HS, closely followed by the ecotypes from S-HS and UL-HS. The minimum of this characteristic was recorded in the ecotypes from DF-SD (**Table 2**; **Figure 2**).

Aerenchyma in the root cortex was recorded in all *Cynodon dactylon* ecotypes, the S-HS ecotype the maximum of this characteristic, which was about 2-folds greater that the second best M-RB. The ecotypes from saline wetlands (KL-HS and UL-HS) also had large aerenchyma in their cortex (**Table 2**; **Figure 2**).

Metxylem area was the maximum in the ecotype from saline wetland UL-HS, which was closely followed by the ecotypes from another saline wetland KL-HS. Two ecotypes from highly salt-affected areas (S-HS and PA-HS) also showed large metaxylem vessels. The S-SW showed the minimum of this characteristic (**Table 2**; **Figure 2**). Three ecotypes (KL-HS, UL-HS and S-SW) surpassed all other ecotypes regarding phloem area, all from highly saline wetlands. The minimum value for phloem area was recorded in two ecotypes, BG-NS and G-SSA.

Pith area was maximum in plants from UL-HS, which was closely followed by that recorded in S-HS, while minimum was recorded in S-SW ecotype. Pith cell area, however, showed slightly different trend and the maximum was recorded in S-HS ecotype that was significantly higher than the second best UL-HS, while the minimum pith cell area was recorded in G-SSA (**Table 2**; **Figure 2**).

## Stem microscopic analysis

**Figure 3:**
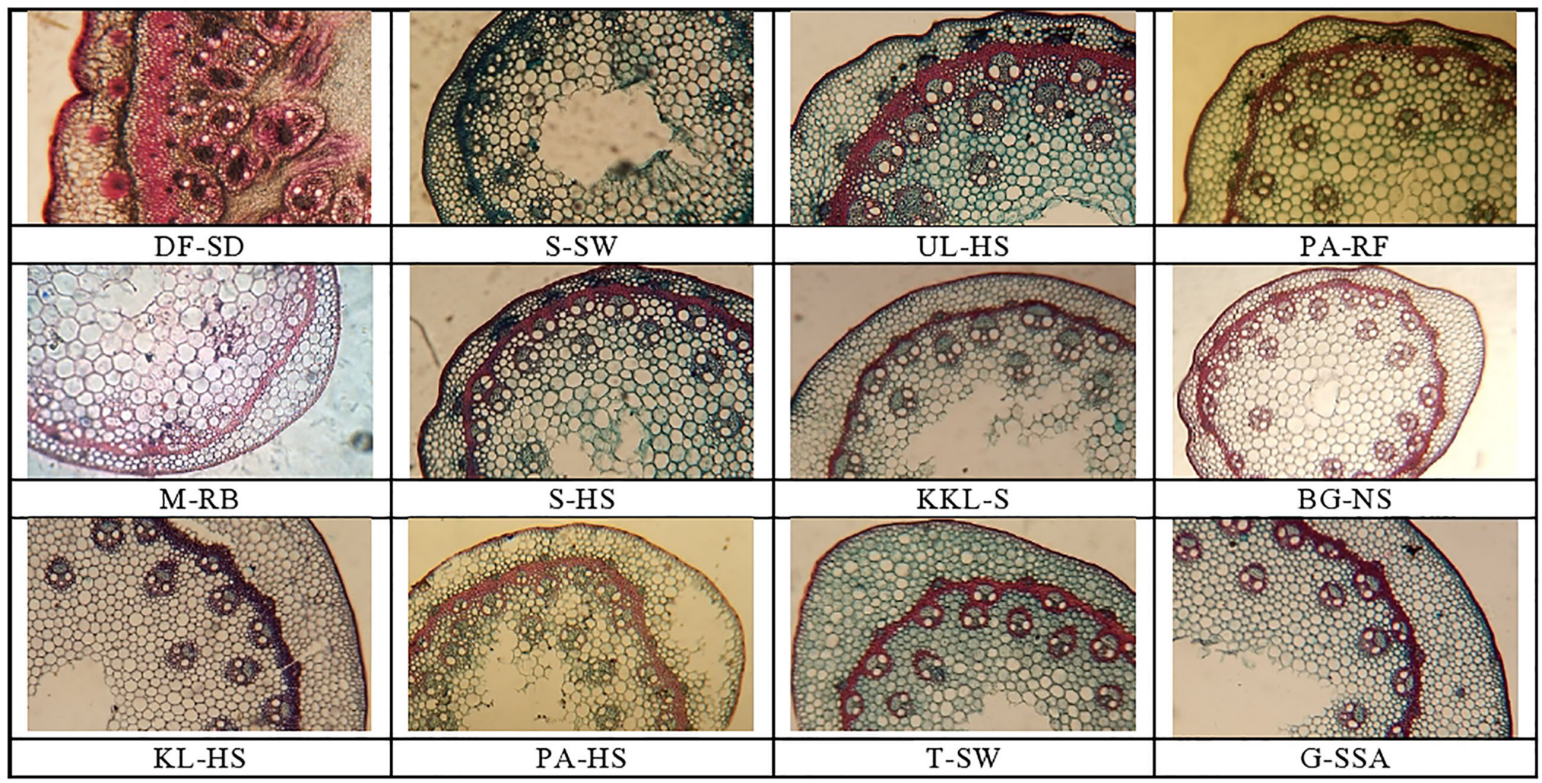
Stem tranverse sections of cynodon ecotypes collected from the Punjab, Pakistan. DF-SD, Derawar fort-saline dessert; M-RB, MUZAFFAR GARH-RIVER BANK; KL-HS, khabbeki lake-hyper saline; UL-HS,Ucchali Lake-Hyper Saline;KKL-S; Kalar Kahar Lake-Saline;T-SW,Treemu-Saline Wetland; S-SW, Sahianwala-Saline Wetland; S-SH, Sahianwala-Hyper Saline; PA-HS, Pakka Anna-Hyper Saline; PA-RF, Pakka Anna-Reclaimed Field; BG-NS, Botanic Garden-non saline; G-SSA, Gatwala-saline semiarid.

Stem area was maximum in BG-NS ecotype, it was followed by that recoded in DF-SD. The minimum stem area was noted in M-RB ecotype. Epidermal cell area was maximum in KL-HS, which was followed by the saline desert habitat F-SD. The minimum value for epidermal cell area was recorded in two ecotypes, M-RB and S-HS (**Table 3**; **Figure 3**).

**Table 3:** Stem anatomical characteristics of the selected habitats from Punjab, Pakistan.

Habitats	Stem area (mm^2^)	Epidermal cell area (µm^2^)	Cortex region thickness (µm)	Cortical cell area (µm^2^)	Sclerenchymatous thickness (µm)	Vascular bundle area (µm^2^)	Vascular bundle number	Metaxylem area (µm^2^)	Phloem area (µm^2^)
DF-SD	11.4g	627.8h	467.8c	6871.0d	326.4i	122073.6h	58.0f	3156.5b	18066.9k
M-RB	5.1a	52.3a	307.4b	7777.8f	65.3c	49352.6f	36.7bcd	5057.3c	7952.2e
KL-HS	10.4f	662.7i	554.9e	8370.8g	114.2g	50834.9f	39.0cde	2860.0a	7394.2d
UL-HS	10.2f	488.3g	484.2d	5580.5c	136.0h	74534.7g	41.0e	6330.4d	17456.5j
KKL-S	8.7e	331.3e	348.2c	6295.5d	46.2a	56380.6g	40.0de	5388.7c	14491.9i
T-SW	6.8c	475.5f	633.8g	14526.8	89.8e	45812.5e	38.0cde	3208.8b	9016.0f
S-SW	5.2a	87.2.7c	310.1b	4255.1a	70.7d	35750.1c	33.0bc	2092.7a	4359.8b
S-HS	7.8d	52.3a	285.6a	5929.3c	54.4b	46562.4f	32.0b	7673.2e	9434.5h
PA-HS	6.3b	244.2b	538.6e	7463.9e	97.9f	33831.8b	42.0e	3348.3b	7376.7d
PA-RF	10.4f	139.5d	296.5a	7132.6e	70.7d	31826.3b	40.0de	5057.3c	5946.7c
BG-NS	11.7g	627.8h	274.7a	4865.5b	46.2a	29210.5a	25.0a	2511.2a	3784.3a
G-SSA	6.8c	87.2f	590.2f	8440.5g	62.6c	40807.5d	33.3bc	3034.4b	9120.6g

DF-SD, Derawar Fort-saline desert; M-RB, Muzaffar garh-River bank; KL-HS, Khabbeki Lake-hyper saline; UL-HS, Ucchali Lake-hyper saline; KKL-S, Kalar Kahar Lake-saline; T-SW, Treemu-wetland; S-SW, Sahianwala-saline wetland; S-HS, Sahianwala-hyper saline; PA-HS, Pakka Anna-hyper saline; PA-RF, Pakka Anna-reclaimed field; BG-NS, Botanic Garden-non saline; G-SSA, Gatwala-saline semiarid.

Cortical region thickness was the maximum in T-SW, which was followed by the cortical thickness in G-SSA and KL-HS. The BG-NS ecotype showed the minimum of this characteristic. Cortical thickness showed a different trend from that recorded in case of cortical region thickness, the maximum was recorded in G-SSA and the minimum in S-SW. Sclerification, however, was more intensive in heavily salt affected habitats, the maximum was recorded in DF-SD, which was more than 2-folds greater than the second maximum in UL-HS. The minimum sclerification was recorded in two ecotypes, BG-NS and KKL-S (**Table 3**; **Figure 3**).

Number of vascular bundles was observed maximum in DF-SD, significantly more than the second best PS-HS. Vascular bundle area and phloem area also showed similar trend, being the maximum in DF-SD. The minimum value for vascular bundle number, as well as vascular bundle area and phloem area was recorded in BG-NS. Wide metaxylem vessels were recorded in the ecotypes from saline lakes, the maximum was recorded in UL-HS, followed by that in KKL-S. The minimum of this characteristic was recorded in S-SW (**Table 3**; **Figure 3**).

## Leaf sheath microscopic analysis

**Figure 4:**
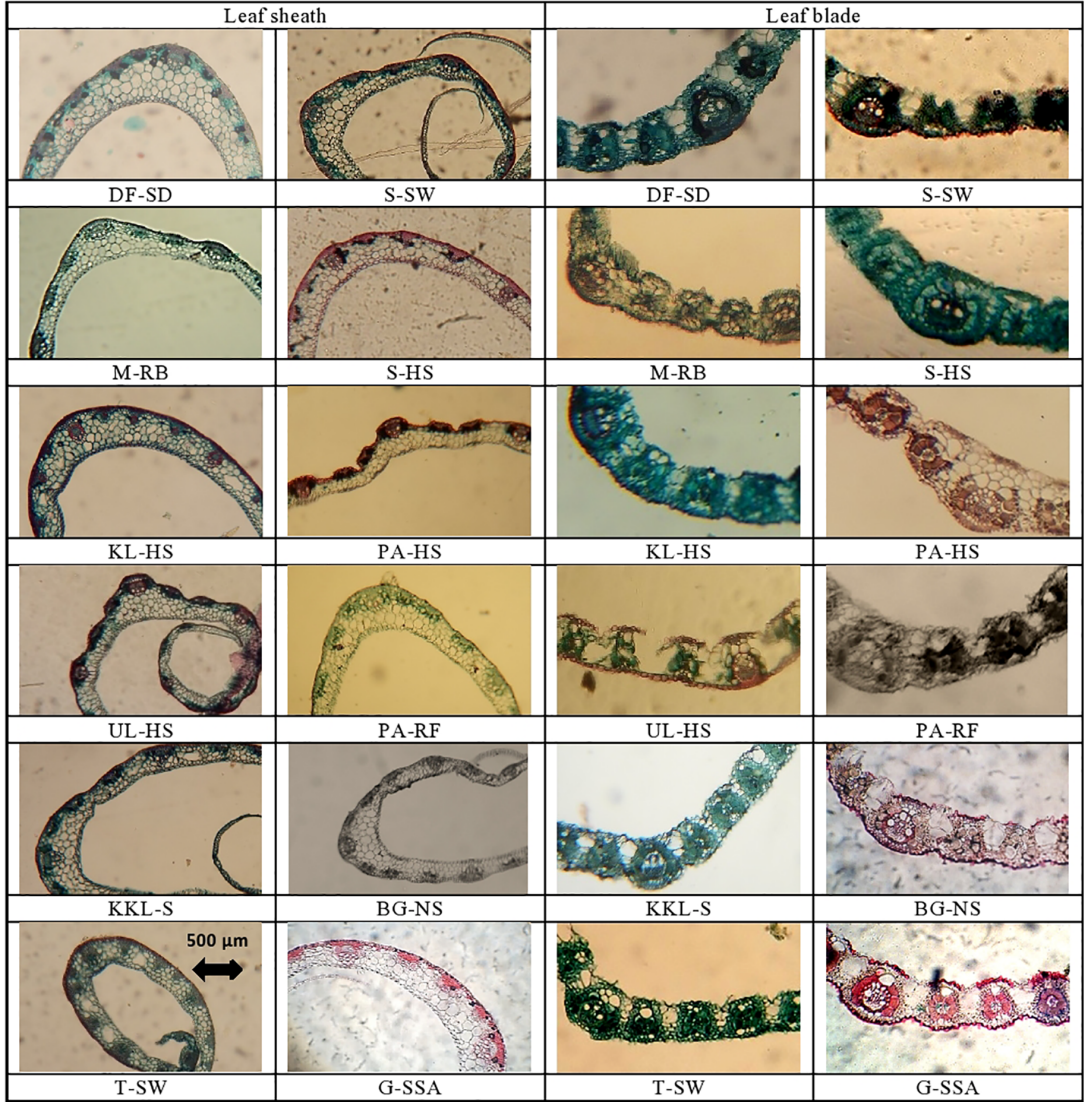
Leaf sheath and leaf blade transverse section of cynodon dactylon ecotypes collected from the Punjab, Pakistan. DF-SD, Derawar fort-saline dessert; M-RB, Muzaffar garh-River bank; KL-HS, Khabbeki Lake-hyper saline; UL-HS, Ucchali Lake-hyper saline; KKL-S, Kalar Kahar Lake-saline; T-SW, Treemu-wetland; S-SW, Sahianwala-saline wetland; S-HS, Sahianwala-hyper saline; PA-HS, Pakka Anna-hyper saline; PA-RF, Pakka Anna-reclaimed field; BG-NS, Botanic Garden-non saline; G-SSA, Gatwala-saline semiarid.

Leaf sheath thickness was the maximum in DF-SD ecotype, which was significantly higher than the second best KL-HS. Thin leaf sheath was recorded in G-SSA, S-HS and T-SW. The maximum value for epidermal cell area was noted in S-HS ecotype, which was followed by that in KKL-S and DF-SD. The thinnest epidermal layer was recorded in plants from PA-RF (**Table 4**; **Figure 4**).

**Table 4:** Leaf anatomical characteristics of the selected habitats from Punjab, Pakistan.

Habitat	Midrib thickness (µm)	Lamina thickness (µm)	Adaxial epidermal cell area (µm^2^)	Abaxial epidermal cell area (µm^2^)	Sclerenchymatous thickness (µm)	Bulliform cell area (µm^2^)	vascular bundle area (µm^2^)	Metaxylem area (µm^2^)	Phloem area (µm^2^)	Trichome density	Trichome length (µm)	Stomatal area (µm^2^)	Stomatal density
DF-SD	693.6e	489.6d	418.5c	627.8d	81.6d	29890.6i	126171.8j	6522.2h	20578.1f	11.0bc	285.6f	16567.1b	14.0cd
M-RB	652.8d	408.0a	470.9d	313.9c	65.3c	6208.3a	99071.5f	4569.0f	28199.0j	11.0bc	184.9b	21293.1d	15.0d
KL-HS	571.2b	522.2c	470.9d	52.3a	122.4f	21345.4e	99350.5f	6993.1i	24589.1h	10.0ab	269.3e	17247.3c	11.0b
UL-HS	571.2b	505.9e	3139.0h	627.8d	81.6d	16113.7b	60565.9a	1290.5a	8597.5a	10.0ab	345.4g	26925.9f	10.0b
KKL-S	554.9a	408.0b	627.8e	1098.7g	97.9e	24065.9f	69058.8b	2005.5b	23595.1g	15.0d	236.6d	27292.2f	10.0b
T-SW	571.2b	408.0b	627.8e	52.3a	40.8a	18311.0d	68064.8c	3505.3e	18938.9e	10.0ab	212.2d	23019.6e	8.0a
S-SW	644.6d	432.5c	209.3a	52.3a	81.6d	26786.4g	84038.9e	5406.1g	15834.7b	17.0e	198.6c	24344.9e	13.0c
S-HS	799.7f	709.9h	1046.4g	313.9c	122.4f	17613.5cd	119143.8i	8266.1j	26298.1i	18.0e	182.2b	34355.0g	10.0b
PA-HS	840.5h	538.6g	889.4f	802.2f	57.1b	17247.3c	115237.4h	2563.0c	17840.2d	9.0a	272.0f	15712.6a	22.00e
PA-RF	824.2g	530.4fg	366.2b	662.7e	146.9g	28634.9h	169159.1k	9033.5k	26751.6i	9.0a	361.8h	33483.0g	13.0c
BG-NS	595.7c	448.8c	627.8e	209.3b	65.3c	51846.4k	102977.8g	5406.1g	16741.5c	12.0c	163.2a	20333.9d	11.0b
G-SSA	628.3d	514.1ef	418.5c	627.8d	81.6d	35575.7j	77917.8d	3173.9d	18328.5e	14.0d	285.6f	24275.2e	11.0b

DF-SD, Derawar Fort-saline desert; M-RB, Muzaffar garh-River bank; KL-HS, Khabbeki Lake-hyper saline; UL-HS, Ucchali Lake-hyper saline; KKL-S, Kalar Kahar Lake-saline; T-SW, Treemu-wetland; S-SW, Sahianwala-saline wetland; S-HS, Sahianwala-hyper saline; PA-HS, Pakka Anna-hyper saline; PA-RF, Pakka Anna-reclaimed field; BG-NS, Botanic Garden-non saline; G-SSA, Gatwala-saline semiarid.

Ecotype DF-SD from a saline desert showed the maximum value for cortical cell area, and this was followed by the cortical cells in PA-RF and G-SSA. The T-SW ecotype had the minimum cortical cell area, which was significantly lower than the second minimum UL-HS. Intensive sclerification was recorded in T-SW and KL-HS ecotypes, whereas the minimum of this characteristic was noted in BG-NS ecotype. Lysogenous cavities (aerenchyma) were observed in only 4 ecotypes, the largest was recorded in T-SW and the smallest in KKL-S. The other two ecotypes, S-HS and KL-HS were from hyper-saline habitats (**Table 4**; **Figure 4**).

The maximum value for vascular bundle area was recorded in plants from G-SSA, which was followed by that in KKL-S and DF-SD. The smallest vascular bundles were recorded in T-SW. Metaxylem area was maximum in PA-RF ecotype, however, the DF-SD ecotype also showed wide metaxylem vessels. The minimum of this parameter was seen in KL-HS ecotype. The DF-SD ecotype surpassed all others regarding phloem area and this was followed by phloem area in G-SSA ecotype. The minimum value of this parameter was recorded in M-RB ecotype from fresh water habitat (**Table 4**; **Figure 4**).

## Leaf microscopic analysis

Midrib thickness was the maximum in PA-HS ecotype, followed by that recorded in PA-RF and S-SH ecotypes. Lamina thickness, on the other hand, was the maximum in S-SH. The minimum value for midrib and lamina thickness was recorded in the KKL-S ecotypes, whereas two other ecotypes, M-RB and T-SW, also possessed the thinnest lamina (**Table 5**; **Figure 4**).

**Table 5:** Leaf sheath anatomical characteristics of the selected habitats from Punjab, Pakistan.

Habitat	Leaf Sheath thickness (µm)	Epidermal cell area (µm^2^)	Cortical cell area (µm^2^)	Sclerenchymatous thickness (µm)	vascular bundle area (µm^2^)	Metaxylem area (µm^2^)	Phloem area (µm^2^)	Aerenchyma cell area (µm^2^)
DF-SD	568.5i	1011.5j	15468.5l	81.6g	35314.2j	1517.2h	10986.6i	0.0a
M-RB	467.5d	802.2f	11701.6i	68.0de	28914.0i	1046.4ef	4446.9a	0.0a
KL-HS	530.4h	523.2c	9068.3d	108.8i	27100.3h	122.1a	5231.7d	14509.3c
UL-HS	522.2h	470.9b	6156.0b	62.6c	23368.4b	1081.2g	4970.1d	0.0a
KKL-S	484.2e	1220.7k	9591.5e	89.8h	36255.9k	1028.9e	7324.4g	13602.5b
T-SW	424.3b	976.6i	3609.9a	136.0j	21258.3a	470.9b	4603.9c	15241.8e
S-SW	497.8f	906.8h	11161.0h	73.4ef	26542.3f	906.8d	8021.9h	0.0a
S-HS	421.6b	1656.7l	10358.8f	76.2fg	25565.7e	470.9b	4586.5b	14910.4d
PA-HS	435.2c	610.4e	6836.1c	57.1b	26786.4g	1081.2g	4935.3d	0.0a
PA-RF	505.9g	279.0a	15224.3k	65.3cd	24292.6c	2075.3i	5580.5e	0.0a
BG-NS	522.2h	592.9d	10777.3g	48.9a	24990.2d	575.5c	5580.5f	0.0a
G-SSA	356.9a	871.9g	13933.8j	89.8h	37930.0l	1063.8fg	10184.4i	0.0a

DF-SD, Derawar Fort-saline desert; M-RB, Muzaffar garh-River bank; KL-HS, Khabbeki Lake-hyper saline; UL-HS, Ucchali Lake-hyper saline; KKL-S, Kalar Kahar Lake-saline; T-SW, Treemu-wetland; S-SW, Sahianwala-saline wetland; S-HS, Sahianwala-hyper saline; PA-HS, Pakka Anna-hyper saline; PA-RF, Pakka Anna-reclaimed field; BG-NS, Botanic Garden-non saline; G-SSA, Gatwala-saline semiarid.

The thickest epidermal layer on adaxial leaf surface was recorded in UL-HS ecotype, while on abaxial surface, thickest epidermal layer was observed in KKL-S ecotype. The minimum value for this parameter was recorded in S-SW ecotype on adaxial side, and in three ecotypes (KKL-S, KL-HS and T-SW) on abaxial side (**Table 5**; **Figure 4**).

Sclerification in leaves was maximum in PA-RF ecotype, followed by, KL-HS and S-HS ecotypes. The T-SW ecotypes showed the minimum value for sclerenchyma thickness. Bulliform cell area was maximum in BG-NS, which was more than 2-folds than the second maximum in G-SSA. The smallest bulliform cells were recorded in the M-RB ecotype.

**Figure 5:**
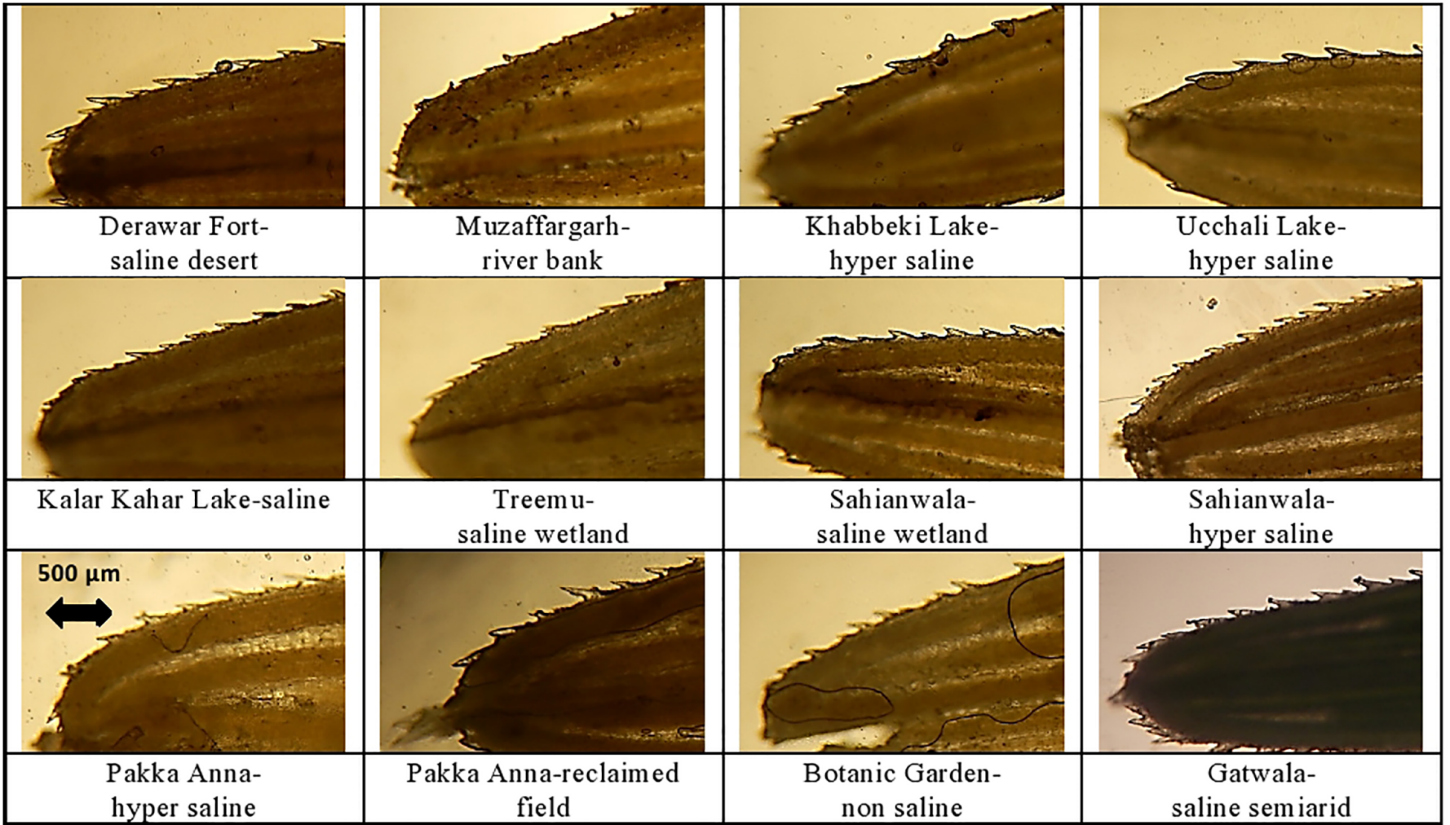
Leaf trichomes and leaf blade transverse section of cynodon dactylon ecotypes collected from the Punjab, Pakistan. DF-SD, Derawar fort-saline dessert; M-RB, Muzaffar garh-River bank; KL-HS, Khabbeki Lake-hyper saline; UL-HS, Ucchali Lake-hyper saline; KKL-S, Kalar Kahar Lake-saline; T-SW, Treemu-wetland; S-SW, Sahianwala-saline wetland; S-HS, Sahianwala-hyper saline; PA-HS, Pakka Anna-hyper saline; PA-RF, Pakka Anna-reclaimed field; BG-NS, Botanic Garden-non saline; G-SSA, Gatwala-saline semiarid.

Vascular bundle area, as well as metaxylem vessel area was the maximum in the PA-RF ecotype and the minimum in the UL-HS ecotype. The DF-SD ecotypes also showed large vascular bundles, whereas wide metaxylem vessels were observed in the S-HS ecotype. The maximum phloem area was recorded in M-RB, followed by that recorded in PA-RF and S-HS. The minimum value for phloem area was observed in UL-HS ecotype (**Table 5**; **Figure 5**).

Leaf trichomes density was maximum in plants inhabiting S-HS site, which was closely followed by that in S-SW and KKL-S. Its minimum value was recorded in two ecotypes, PA-HS and PA-RF. Trichome length, in contrast, was maximum in PA-RF and minimum in BG-NS ecotype (**Table 5**; **Figure 5**). Stomatal density was maximum in S-HS, while maximum stomatal area was recorded in PA-HS ecotype. The minimum value for stomatal density and stomatal area was recorded in T-SW and PA-HS ecotype respectively (**Table 5**; **Figure 5**).

## Specific anatomical modifications

In roots, the S-HS ecotype showed exceptionally large aerenchyma along with large endodermis. A reverse was recorded in the DF-SD ecotype, which had very little proportion of cortex, as well as aerenchymatous region (**Figure 2**). The ecotype from KL-HS showed a unique modification, where one side is with large aerenchyma and the other is with intact closely-packed parenchyma. Two ecotypes, S-SW and G-SSA, showed intensive sclerification in the vascular region, but that from saline semi-arid region also showed sclerification in the pith.

A very unusual modification in DF-SD ecotype is the development of large-sized vascular tissue in the stem, which are located in 4-5 rings, not only near the stem periphery but also inside the sclerenchymatous ring inside cortical region (**Figure 3**). Pith is restricted to a small central region only, whereas vascular bundles occupy a large proportion in the stem. Moreover, vascular region is also densely sclerified. The UL-HS ecotype also possessed numerous large vascular bundles, but these bundles are not closely packed as noted in the DF-SD ecotype. These bundles are sclerified only at outer region; however, sclerenchymatous ring is very well developed in this ecotype.

The thickest leaf sheath was recorded in the DF-SD ecotype, comprising of significant parenchymatous region. Two ecotypes, PA-HS and UL-HS are with wavy outer surface of the leaf sheath. Vascular bundles are near the periphery, but intensively sclerified at outer region. Parenchymatous region is in grooves in both ecotypes (**Figure 3**).

An exclusive feature of the ecotype PA-HS is the presence of storage parenchyma in leaf midrib, which has not been recorded in any other ecotype studied in the present investigation. Microhairs have also been recorded on the adaxial leaf surface in *C. dactylon* ecotypes, but their density was exceedingly high in the G-SSA ecotype (**Figure 3**).

## RDA analysis

**Figure 6:**
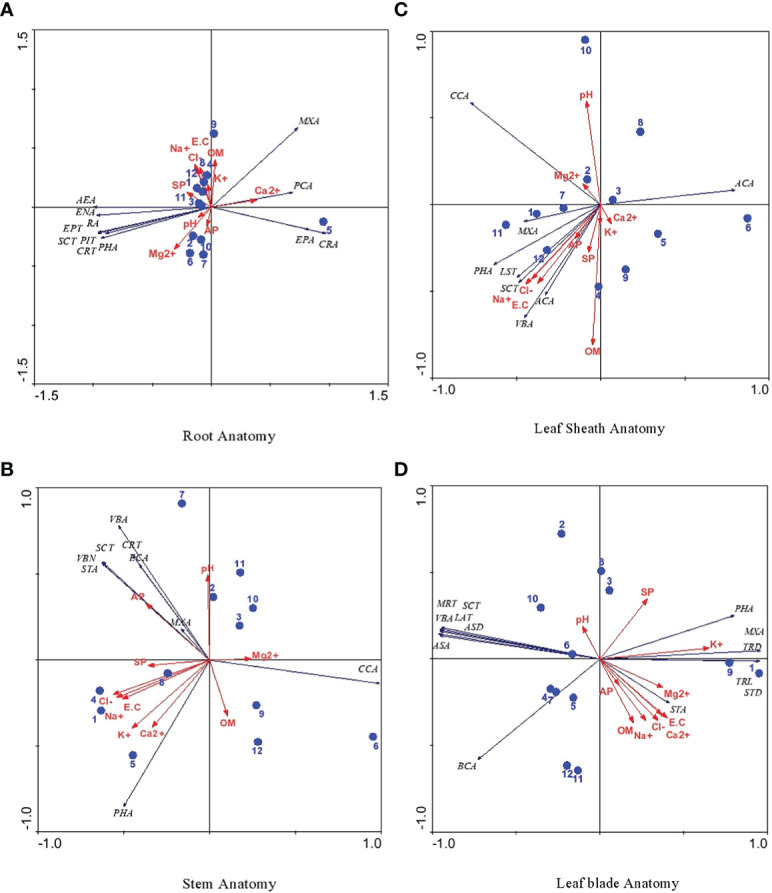
**(A-D)**: RDA ordination triplot showing correlation of soil physico-chemical, anatomical characteristics of cynodon dactylon ecotypes collected from the Punjab, Pakistan. 1 Derawar Fort-Saline Desert, 2 Muzaffar Garh-River Bank, 3 Khabbeki Lake-Hyper Saline, 4 Ucchali Lake- Hyper Saline, 5 Kalar Kahar Lake-Saline, 6 Treemu-Saline Wetland, 7 Sahianwala-Saline Wetland, 8 Sahianwala-Hyper Saline, 9 Pakka Anna-Hyper Saline, 10 Pakka Anna-Reclaimed Field, 11 Botanic Garden-Non Saline, 12 Gatwala-Saline Semiard, Leaf anatomical characteristics are abbreviated as LBT, Leaf Blade Thickness; SCT, Sclerenchymatous Thickness; BCA, Bulliform Cell Area; VBA, Vascular Bundle Area; MXA, Metaxylem Area; PHA, Phloem Area; TRL, Trichome Length; TRD, Trichome Density; ASA, Abaxial Stomatal Area; ASD, Abaxial Stomatal Density; ADA, Adaxial Stomata Area and ADD, Adaxial Stomatal Density.

The RDA ordination triplot of root characteristics showed a strong association of KKL-S with epidermal cell area and cortical cell area (**Figure 6A**). Pith cell area was correlated with soil Ca^2+^, whereas organic matter and K^+^ were associated with DF-SD, UL-HS and PA-HS. Soil Mg^2+^ was associated with M-RB and available P with PA-RF and S-SW. Soil ECe, Na^+^ and Cl^-^ were strongly correlated with G-SSA, S-HS and UL-HS sites. The other soil or anatomical characteristics showed either no or weak correlation with study sites.

Among stem anatomical characteristics, only available P was strongly associated with stem cross-sectional area, vascular bundle number and sclerenchymatous thickness, whereas other characteristics showed no correlation at all. Leaf sheath characteristics like leaf sheath thickness and sclerenchymatous thickness showed a strong influence of soil Na^+^, Cl^-^, ECe and available P. Soil Mg^2+^ was strongly correlated with cortical cell area, whereas metaxylem area was associated with BG-NS, DF-SD and S-SH sites (**Figure 6B**).

RDA for leaf blade and leaf sheath anatomical characteristics showed a strong association of T-SW with midrib and lamina thickness, sclerenchymatous thickness, vascular bundle area and adaxial and abaxial epidermal cell area (**Figures 6C, D**). Metaxylem area and trichome density were influenced by soil K^+^, whereas UL-HS and S-SW sites were associated with bundle sheath cell area. Stomatal area showed a weak correlation with soil Mg^2+^.

## Discussion

*Cynodon dactylon* is a C_4_ perennial grasses (Kumar et al., 2011) that can survive under a variety of habitats including high salinity (Yin et al., 2022), waterlogging (Xie et al., 2015) and arid and semi-arid conditions (Malik et al., 2015). Since evolution plays a critical role in evolving tolerant ecotypes, investigation differently adapted ecotypes have been found for their differential structural adaptation to cope environmental extremes (Polle and Chen, 2015). Key mechanisms that have been identified as mediating stress tolerance well be discussed at the scale of anatomical level, to understand how to plants survive in adverse environments.

The most critical impact of environmental stresses like salinity and drought is related to water scarcity, and therefore conservation of water is the utmost requirement of a plant species. The first line of defense is the restriction of water loss through plant surface (Toon et al., 2015) and it is more important in above-ground plant parts (Fathi and Tari, 2016). Majority of the ecotypes were collected from moderately to highly saline sites, where soils are more or less compact, especially in the dry land salinities. It is therefore, expected that roots are more prone to damage due to soil friction. In that scenario, epidermis and/or cortical region inside is critical, because intact tissues can resist soil friction and compactness (Zörb et al., 2015) and hence they are can survive under adverse environments (Parida et al., 2016). Ecotype S-HS from hard saline/sodic soil with thick epidermal layer and outer cortical may ensure its survival.

Water is a rare commodity for arid or saline arid regions, and under such circumstances resistance to evaporation loss can be a major factor for the survival of a plant species. As an important phenomenon for water conservation, plants adapted to water scarce environments are generally equipped with dense cuticle and larger lignified epidermis which minimizes water loss (Dörken et al., 2020). In this regard, the stem of *C. dactylon* is prostrate and horizontally spreading on the ground (Nitu et al., 2019). Moreover, a significant proportion of leaf sheath covers the lower part of stem, and hence any modification in leaf sheath (or leaf blade) that is involved in water loss must be critical (Nitu et al., 2019, Polle and Chen, 2015).

Leaves in this species are with deeply inserted bulliform cells, which enable leaf to roll, and protecting adaxial epidermis and stomata from a direct exposure to hot and dry external environments (Matschi et al., 2020). However, larger bulliform cells were observed in the BG-NS ecotype collected from the non-saline and well irrigated region. Therefore, it can be concluded that moisture availability is crucial for leaf development as well as bulliform cells (Hameed et al., 2014). Besides, presence of bulliform cells in thin fibrous (with more sclerenchyma) leaves like those in the DF-SD ecotype is of significant ecological importance than enables leaf to roll efficiently (Grigore et al., 2010).

Presence of trichomes on the leaf surface, as well as on leaf margin is a characteristic feature of desert/saline desert plants that can contribute significantly towards water conservation (Naz et al., 2016). To cope with high saline conditions, DF-SD ecotype had long and dense hairiness. Moreover, the tolerance of the two other ecotypes from saline to hyper-saline habitats (S-HS and G-SSA) can be explained by their leaf marginal hairs. Another type of hairs i.e., microhairs have also been recorded in many *C. dactylon* ecotypes which are known as salt excretory (Farooq et al., 2015; Li et al., 2015). These microhairs are more prominent and dense in the ecotypes from saline arid areas like G-SSA and DF-SD. In this regard, improved salt excretion by such microhairs has also been reported in many salt tolerant or halophytic species (Askary et al. (2016); Rajakani et al. (2019) in *Mentha piperta* and Céccoli et al. (2015). In addition to microhairs, storage parenchyma in water conservation is immensely important. This can explain why desert and halophytic perennials are generally succulent (Favaretto et al., 2015). Root cross-sectional area in *C. dactylon* depends on the proportion of parenchymatous tissue (cortex and pith) that is mainly involved in storage of water (Chimungu et al., 2015). In addition, involvement of arenchymatous tissue in salt tolerance (Rossatto et al., 2015) has also been widely reported in halophytic species like stem-succulent halophytes *Tecticornia pregranulata* (English and Colmer, 2011), *Fimbristylis dichotoma* (Hameed et al., 2012), *Lasiurus scindicus* (Naz et al., 2015) and *Juncus* species (Al Hassan et al., 2015). Here, a well-developed aerenchyma in cortical region is a distinctive feature of roots in *C. dactylon*, which is involved in gaseous exchange in hydrophytes (Lacramioara et al., 2015). Stems are partially covered by leaf sheath in *C. dactylon*, and hence storage parenchyma is very much protected from water loss (Shamah et al., 2019). Moreover, extended proportion of storage parenchyma provides additional space to retain moisture in the stem tissue (Makbul et al., 2011). It was observed that ecotypes from hyper-saline lakes (UL-HS and KL-HS) had greater percentage of parenchyma in their stem, besides central lysogenous cavities, which support it to survive in saline waterlogged non-aerated environments (Joshi and Kumar, 2012). Presence of large amount of parenchymatous tissue in leaf sheath is of great ecological significance that provides extra space for water storage. The DF-SD ecotype from saline desert require significantly more storage tissue to acclimatize extreme water deficit climatic conditions, specifically the organs like leaf sheath that are more exposed to external environments. Storage parenchyma in leaf midrib has only been recorded in the PA-HS ecotype, and this characteristic confirms its better performance in extreme salinity (Paramonova et al., 2004). Moreover, aerenchyma also aids in bulk salt movement in excretory halophytes (Zhou et al., 2015). Therefore, presence of root aerenchyma can be related to ecological success of *C. dactylon* in a variety of environments. In our studies, the ecotypes from heavily salt affected-areas like S-HS and UL-HS not only had larger parenchymatious region but also aerenchyma in roots.

Another trait of prime importance for living in saline arid condition, where water conservation is the first priority of a plant is sclerification of soft and delicate tissue like vascular tissues (Ola et al., 2012). Sclerifitaion not only provides mechanical strength to parenchymatous tissues and preventing them from collapse but also prevents water loss (Cholewa and Griffith, 2004), particularly from aerial plant organs. The DF-SD ecotype from extreme saline desert showed an exceptional alteration of vascular tissues, which occupies about 60% of the total stem area and are closely packed and intensively sclerified. *Cynodon dactylon* is mainly propagated by the horizontal stems (stolon).

Another characteristic feature of a stem in *C. dactylon* is the presence of sclerenchymatous ring inside stem periphery, which encircles vascular tissues. This ring is more developed in ecotypes from high salinities (S-HS, KL-HS and UL-HS). Sclerenchymatous protection outside vascular tissue resists radial water movement in stem (Hameed et al., 2010) and therefore is vital for water conservation under water shortage. Leaf sheath is also variably sclerified on the outer side of vascular bundles, but the ecotypes PA-HS and UL-HS showed distinct grooves in leaf sheath, protecting parenchymatous region from external environments such as wind (Machado et al., 2015).

Bullifrom cells are of great ecological significance, as they are involved in leaf rolling (Zhang et al., 2015), hence protecting stomata (which are only recorded on the abaxial side) in a tight cylinder. It is an imperative strategy of plants dominating saline arid environments. Such modification minimizes transpiration rate significantly, and consequently enhancing water use efficiency (Chen et al., 2015). Bulliform cells when become turgid due to water storage, this water can be utilized in other metabolic functions vital for the survival of a plant.

Last but not the least, efficiency in conduction of solutes is meaningful, especially in environment where conservation of water is prime strategy for the survival of a plant. Exceptionally large and numerous vascular bundles in the ecotypes DF-SD and UL-HS, that were collected from extreme salinities, is an indication of their importance for salinity tolerance. A positive relation of vascular tissue with efficiency of conduction has earlier been reported by (De-Rybel et al., 2016) and Elhalim et al. (2016).

Each ecotype showed different type modifications for their existence in a specific habitat that reflects to their ecological success. DF-SD ecotype is exposed to multiple stress like high salinity, extreme aridity and heat. These conditions induced modifications like stem cross-sectional area and leaf sheath thickness that contributes towards water storage, vascular tissues for proficient translocation of solutes and trichomes. Consequently, this provides resistance to water loss, and hence water conservation is the first priority (Polle and Chen, 2015). Another ecotype G-SSA from saline arid habitat where rainfall is not as low as in the Cholistan Desert, sclerification in root is the only notable modification. The two ecotypes from hyper saline wetlands, UL-HS and KL-HS relied on the number and size of vascular tissue, central cavity and sclerification in stem that is important for conduction of solutes, water loss and bulk movement of salts, respectively. The ecotype from reclaimed field was not much different from its counterpart from hyper-saline dryland, and this might be due to too short time to be required for the evolution of any specific characteristic. In contrast, the ecotypes from non-saline habitats, M-RB and BG-NS showed no visible sclerification in their above or below ground plant parts and no other prominent modification.

## Conclusions

It is concluded that anatomical characteristics represent adaptive components in *C. dactylon* ecotypes that ensure growth, survival and ecological success against environmental hazards. T he main strategy of most ecotypes was to maintain water conservation, either *via* prevention of water loss or by storing water in the metabolically active plant tissues. On the other hand, ecotypes dominating hyper-saline habitats showed salt excretion by excretory hairs of bulk salt movement in aerenchyma. sclerification in root is the only notable modification in the G-SSA ecotype from saline arid habitat where rainfall is not as low as in the Cholistan Desert. However, sclerification was not observed in M-RB and BG-NS in their above or below ground plant parts and no other prominent modification. Overall, key mechanisms that have been identified as mediating stress tolerance well be discussed at the scale of anatomical level, to understand how to plants survive in adverse environments.

## Data availability statement

The original contributions presented in the study are included in the article/supplementary material. Further inquiries can be directed to the corresponding author.

## Author contributions

AT, experimentation and research design; FA, research design, supervision, validation; MH, research design and validation; MA, statistical analysis and drafting; MKO, research validation and writing; UHS, statistical analysis; NK, validation and research design; MR, writing and validation; AAS, review and drafting; MMH, review and validation; HA, writing and drafting. All authors contributed to the article and approved the submitted version.
